# Biomolecular and Biohybrid Systems for Solar Energy Conversion

**DOI:** 10.3390/ijms24097794

**Published:** 2023-04-25

**Authors:** Joanna Kargul, Margot Jacquet

**Affiliations:** Solar Fuels Laboratory, Centre of New Technologies, University of Warsaw, Banacha 2C, 02-097 Warsaw, Poland

The depletion of fossil fuels and increased amount of atmospheric/environmental pollution associated with the excessive use of fossil fuels to power our economies have intensified the efforts of academia and industry worldwide to seek sustainable technological solutions to meet the global energy demand. Among all the renewable energy resources available on Earth, solar light is by far the most abundant and durable primary source of energy. Indeed, natural photosynthesis, which evolved over 3.5 billion years ago, provides an elegant blueprint of solar-driven conversion technologies dubbed as artificial photosynthesis. Inspired by the molecular principles of natural photosynthesis, solar-converting technologies have emerged as a promising solution to provide not just green electricity, but also energy vectors in the form of solar fuels and chemicals. This Special Issue on “Biomolecular and Biohybrid Systems for Solar Energy Conversion” aims to provide an up-to-date compendium on the recent advances and challenges in the direct solar conversion field, focusing on the recent progress towards the development of rational strategies to achieve the highest power conversion efficiency.

For the biomolecular solar conversion technologies to become viable, it is of paramount importance to understand and investigate the current limitations of these systems. Whilst the structure and function of photosynthetic macromolecular machines have been optimized through billions of years of evolution, their optimal implementation as efficient biocatalysts remains to be realized. This challenge was addressed by the comprehensive and rational nano-engineering of the biophotocatalyst and the catalyst/electrolyte interface described in the paper by Szewczyk et al. [1]. By combining photocurrent measurements with transient absorption spectroscopy analyses, the authors provided an in-depth dissection of the electron transfer process limitations that occur within the model biophotocatalyst, Photosystem I (PSI), upon its partially oriented immobilization on conductive glass (fluorine-dopped tin oxide, FTO). This work has shown the crucial importance of proper photoelectrode design in order to avoid the (quasi)permanent inactivation of photo-oxidized PSI, as well as to minimize the fast intramolecular charge recombination processes, both of which hamper the overall quantum efficiency of the biomolecular solar-converting devices.

In addition to the optimal bio-photoelectrochemical cell configuration, other important considerations include applying the principles of sustainability, cost-effectiveness and use of environmentally friendly materials in the biomolecular system’s design. Indeed, commonly used strategies to improve the interfacial electron transfer in biohybrid electroactive systems rely on the use of large amounts of toxic redox mediators, as well as expensive conductive substrates based on noble and rare elements. To address these design limitations, the groups of B. D. Bruce and C. Villarreal have collaborated to develop a new class of optimized biohybrid dye-sensitized solar cells (BSSCs) [2]. By using the innovative universal counter-electrode based on poly (3,4-ethylenedioxythiophene) (PEDOT) modified with multi-walled carbon nanotubes (PEDOT/CNT), and aqueous bipyridine cobalt^II/III^ complexes as direct redox mediators, they improved the power conversion efficiency in two types of BSSCs with two different bio-photosensitizers, PSI and bacteriorhodopsin. Such a universally biocompatible system showed stable performance over a period longer than two weeks.

The long-term stability of biohybrid solar-converting systems is a crucial aspect that must be addressed in order to compete with the current silicon-based photovoltaic technologies. Biological photosensitizers often suffer from photodamage (photobleaching) through prolonged irradiation exposure, in particular under aerobic conditions. In this context, Szalkowski et al. [3] investigated the wavelength-dependence of the PSI photobleaching dynamics upon immobilization of this biophotocatalyst on a glass substrate in conjunction with the effect of plasmonically active nanostructures, i.e., silver island film (SIF), on the stability and yield of photocurrent generation. The work highlighted the correlation between the photobleaching of the PSI-associated chlorophylls under high light intensity in the presence of oxygen, both of which are suppressed under oxygen-free conditions. Importantly, the authors showed the beneficial effect of the plasmonic interaction between the fluorophores of PSI and SIF on the improved stability of the photo-induced charge-separated state within the PSI biophotocatalyst under anoxic conditions. The work, therefore, paves the way for the design of much more efficient biosolar systems with prolonged photostability and highly improved light harvesting functionality.

Theoretical predictions that arise from quantum mechanical/molecular mechanics calculations can provide valuable information to guide the rational design of the biosolar systems, notably via de novo modeling of various molecular interfaces employed to electrically wire biophotocatalysts in an oriented and well-controlled manner on various types of conductive surfaces. Thus, Kaźmierczak et al. [4] studied a metalorganic interface chemisorbed on single-layer graphene and exacerbated the importance of the molecular composition of such an interface, focusing on the type of metallic redox centers and the degree of saturation of the chemical backbone of the molecules constituting the conductive interface. Such a theoretical approach can aid the experimental fine-tuning of the efficacy and directionality of electron transfer.

Last but not least, a highly promising strategy was described in the article by Izzo et al. [5] to significantly improve PSI biocatalyst loading, provide its uniform orientation with respect to the electrode surface (single-layer graphene on FTO) and simplify the overall configuration of the solar-converting device. The authors achieved these goals by applying the power of innovative genetic engineering to introduce a His_6_ affinity tag on the specific structural domain of the highly robust PSI complex, which aided the strong and oriented binding of this biophotocatalyst to the appropriately functionalized graphene surface. This approach yielded a spectacular improvement of the electron transfer process while maintaining the simplicity of the system’s configuration and easiness of the device manufacturing.

We would like to thank all the authors who have contributed to this Special Issue. We are convinced that this Special Issue will provide an excellent reference to the most recent advances in the biomolecular solar conversion field and will inspire the community to develop efficient and rational strategies for the much-needed significant improvement of biosolar systems’ performance.
